# Volatility Dynamics of Non-Linear Volatile Time Series and Analysis of Information Flow: Evidence from Cryptocurrency Data

**DOI:** 10.3390/e24101410

**Published:** 2022-10-02

**Authors:** Muhammad Sheraz, Silvia Dedu, Vasile Preda

**Affiliations:** 1 Department of Mathematical Sciences, Institute of Business Administration, The School of Mathematics and Computer Science, Karachi 75270, Pakistan; 2 Department of Financial Mathematics, Fraunhofer ITWM, 67663 Kaiserslautern, Germany; 3 Department of Applied Mathematics, Bucharest University of Economic Studies, 010734 Bucharest, Romania; 4 Faculty of Mathematics and Computer Science, University of Bucharest, Academiei 14, 010014 Bucharest, Romania; 5 “Gheorghe Mihoc-Caius Iacob” Institute of Mathematical Statistics and Applied Mathematics of Romanian Academy, 2. Calea 13 Septembrie, nr. 13, Sect. 5, 050711 Bucharest, Romania; 6 “Costin C. Kiritescu” National Institute of Economic Research of Romanian Academy, 3. Calea 13 Septembrie, nr. 13, Sect. 5, 050711 Bucharest, Romania

**Keywords:** volatility, transfer entropy, mutual information, flow of information, financial time series

## Abstract

This paper aims to empirically examine long memory and bi-directional information flow between estimated volatilities of highly volatile time series datasets of five cryptocurrencies. We propose the employment of Garman and Klass (GK), Parkinson’s, Rogers and Satchell (RS), and Garman and Klass-Yang and Zhang (GK-YZ), and Open-High-Low-Close (OHLC) volatility estimators to estimate cryptocurrencies’ volatilities. The study applies methods such as mutual information, transfer entropy (TE), effective transfer entropy (ETE), and Rényi transfer entropy (RTE) to quantify the information flow between estimated volatilities. Additionally, Hurst exponent computations examine the existence of long memory in log returns and OHLC volatilities based on simple R/S, corrected R/S, empirical, corrected empirical, and theoretical methods. Our results confirm the long-run dependence and non-linear behavior of all cryptocurrency’s log returns and volatilities. In our analysis, TE and ETE estimates are statistically significant for all OHLC estimates. We report the highest information flow from BTC to LTC volatility (RS). Similarly, BNB and XRP share the most prominent information flow between volatilities estimated by GK, Parkinson’s, and GK-YZ. The study presents the practicable addition of OHLC volatility estimators for quantifying the information flow and provides an additional choice to compare with other volatility estimators, such as stochastic volatility models.

## 1. Introduction

### 1.1. Complex Systems and Statistical Relationships

In the study of complex systems, statistical relationships such as correlation-based techniques have been extensively used to investigate linear dependence. The most common statistical methods exclusively rely on second-order statistics, such as correlation analysis and Principal Component Analysis (PCA). For example, Granger causality (GC) [1] is based on second-order statistics and focuses on correlation, which constrains its relevance to only linear systems. As a matter of fact, due to the highly increased evidence of nonlinear trends in financial time series analysis, researchers have drawn significant attention to the use of the econophysics framework, such as wavelet transforms, mutual information, correlation dimension test, Hurst exponent, Lyapunov exponent, and information-theoretic measures. For instance, Chu et al. used the wavelet decomposition method and nonlinear causality testing based on Taylor series approximation and investigated bi-directional nonlinear causality between stock returns and investor sentiment in China [2]. Zhao et al. proposed a new mutual-information matrix analysis to study the nonlinear interactions of multivariate time series and revealed the interactions among the individual stocks [3]. The rolling window Spearman correlation and wavelet coherence have been employed to study the dynamic relationships between stock markets [4]. The long and short nonlinear dynamics in Moroccan family business stock returns were studied using stationary wavelet transform, Hurst exponent, and largest Lyapunov exponent methods to investigate the presence of both fractal and chaos in their trend and instant variations [5]. Some studies use applications of non-parametric complexity measures and information-theoretic measures for financial time series. For example, Hurst exponent, Kolmogorov complexity, Lempel–Ziv complexity, and Shannon entropy have been used to compute long memory, information content, information generation, and randomness in various industrial sectors from the Casablanca Stock Exchange (CSE) and Dow Jones and S&P500 [6]. In a study of the unidirectional causal relationship of cryptocurrencies with the COVID-19 pandemic, Toda and Yamamoto (linear) and Diks and Panchenko (nonlinear) Granger causality tests confirm the existence of unidirectional causality [7]. Ghorbel et al. uncovered asymmetric correlations between the stock indexes and cryptocurrencies in both the short and long run and concluded that most stock prices respond more to the negative shocks of cryptocurrencies than to the positive ones [8]. Moreover, Tong et al. examined cryptocurrency price volatility changes over time and are positively correlated. Additionally, Bitcoin has fractal characteristics, and cryptocurrency price fluctuations have a long memory of price volatility. The price fluctuation of cryptocurrency does not follow the random walk model and is a system with chaotic characteristics [9]. In literature, information-theoretic measures frameworks have been significantly used to detect the nonlinear dynamics of asset prices, volatilities, and construction of risk-neutral densities. See, for example, dynamics of volatility and randomness of the Pakistan Stock Exchange (PSX-100), analysis of randomness in estimated GARCH volatilities, return series, and closing prices using Shannon’s, Tsallis, approximate and sample entropies, and construction of risk-neutral densities in [10,11].

### 1.2. Transfer Entropy and Mutual Information

We can identify that several methods to examine the interactions between complex processes have been developed. For example, Causality among variables, events, or objects has been the fundamental question in the literature that can be understood as a “flow” among processes and expressed and analyzed in mathematical language [12]. For instance, mutual information entropy explores only the statistical dependence between two random variables, whereas a non-parametric method for measuring causal information transfer between systems was proposed by Schreiber to demonstrate the bi-directional flow [13]. Following the concept of transfer entropy, Marschinski and Kantz introduced effective transfer entropy and attempted to measure the information flow between the time series data of Dow Jones and the DAX stock index [14]. Indeed, the concept of TE expands to introduce effective transfer entropy (ETE) and Rényi transfer entropy (RTE). The former method improves TE by reducing the influence caused due to noise, and RTE uses different values of its parameter *r* to capture the effect of distinguished probability intervals. In the study of financial markets such as stock or asset returns, tail events usually refer to relatively large positive or negative returns. If these events are more relevant, Rényi transfer entropy provides a tool to give more weight to their contribution to the overall information flow. The TE methods are based on a model-free approach to measuring the effective-directional information flow without the restriction of linear systems. Therefore, mutual information (MI) and bi-directional information flow based on transfer entropy (TE) methods have received considerable attention from researchers. For instance, Baek et al. investigated the strength and the direction of information transfer in the US stock market and found energy industries influence the whole market [15]. Kwon and Yang investigated the directionality of information transfer in US stocks. The results of the study uncovered that individual stocks are influenced by the index of the market. Similarly, the most prominent source of information flow is the USA, while most receivers are in the Asia/Pacific region [16,17]. Information transmission during overlapping hours of the USA and European stock markets during the financial crises of 2007–2008 is also a subject of the study by Dimpf and Peter. The study shows significant bi-directional information transfer between the US and the European markets with a dominant flow from the US market [18]. Using transfer entropy, one can quantify the information flow from the market to the individual stocks to determine the extent of sensitivity to information flow. For example, bi-directional information flow between exchange rates and stock markets exists during the financial crisis of 2008 [19]. Similarly, market expectations and investor fear have been investigated using Renyi entropy and estimated mutual information between return time series of Bitcoin, S&P500, WTI, Brent, Gas, Gold, Silver, and investor fear index represented by VIX in [20]. Unlike Shannon’s transfer entropy, where the information flow between two stochastic processes takes into account the whole underlying empirical price distribution, the Renyi transfer entropy (RTE) describes the information flow only between certain pre-decided parts of two price distributions involved. For example, Jizba et al. show that information also flows from European markets to the US, which is particularly seen from a careful analysis of Rényi information flow between the DAX and S&P500 indices [21]. In an empirical application, Dimpfl and Peter examine the importance of the credit default swap market relative to the corporate bond market for the pricing of credit risk using the Shannon transfer entropy and the effective transfer entropy approaches [22]. Leonidas studied the causal relationships between the 197 largest companies in the world in terms of market capitalization using the effective transfer entropy method to explore the structure of a global network of financial companies [23]. Given the results of the above-discussed studies, the transfer entropy methods have outperformed to quantify the bi-directional flow of information. See more details on the application of Shannon transfer entropy, effective transfer entropy, and Rényi transfer entropy in [24].

Transfer entropy measures the directionality of a variable with respect to time. Recently, some methods have been introduced that do not depend explicitly on time. For example, a permutation-based measure called inner composition alignment (IOTA) due to Hempel et al. contributes to the study of coupling between very-short time series and does not depend explicitly on time [25,26]. Wang et al. proposed to divide the whole series into several short segments to investigate local coupling for long-time series. The study explores interactions between stock market indices and found the interaction between Chinese stock markets is more substantial than that of American ones [27]. Additionally, permutation entropy (PE) is another information-theoretic measure that uses permutation patterns to infer the complexity of time series. The PE method finds linear and nonlinear dependence without imposing any constraints on the theoretical probability distribution of data [28]. Shi et al. [29] introduced an information-theoretic measure named cross-permutation entropy (CPE), inspired by the permutation entropy-based processes. The novel approach is used to detect the cross-correlation between two synchronous time series. The results of the study show that the transfer entropy (TE) method outperformed figuring out the interaction direction between stock markets. However, CPE performs perfectly in the analysis of cross-correlation between stock markets. Bivariate approaches, such as GC and TE, may suffer from shortcomings, such as omitted variable bias that can lead to erroneous conclusions. In an empirical study, Papana and Papana-Dagiasis introduced different variants of partial transfer entropy (PTE) to show the superiority of the suggested variants over TE and PTE [30]. Additionally, the transfer entropy method relies on past observations of the processes. Its numerical estimation requires two factors: (i) the embedding technique and (ii) the entropy estimator. In literature, many entropy estimators exist with different assumptions, limitations, and advantages. Therefore, the choice of the best method to estimate TE for each specific application is still an open problem. For instance, Rozo et al. studied five different estimation methods, and the results suggest the adaptive partitioning method outperformed [31]. However, the literature emphasizes that for highly nonlinear and non-Gaussian data, for example, in our case of cryptocurrency data, it is better to approach causality using the TE information method instead of the traditional Granger causality test [32].

### 1.3. Cryptocurrencies and Transfer Entropy

Since 2009, numerous cryptocurrencies have been developed (CoinMarketCap, 2019). In the list of cryptocurrencies, Bitcoin is the most popular, representing the highest market cap with rapid and continuous price fluctuations. The change in Bitcoin (BTC) prices generally affects other digital currencies. Therefore, investigating the linkage between time series datasets of BTC and other cryptocurrencies could reveal many fascinating properties to enquire about the impact of BTC on other selected digital currencies. For example, Bitcoin sometimes exhibits an increase(decrease) of tens of thousands of dollars in a single day. Several studies have shown that the Bitcoin price is highly volatile compared to traditional fiat currencies [33]. Chen et al. studied the statistical properties of Bitcoin and other cryptocurrencies and investigated those returns are non-normal. However, no single distribution fits well jointly with all the cryptocurrencies analyzed [34]. Cryptocurrency returns mostly deviate from the normality assumption and follows fat-tailed distributions [35]. Chu et al. studied the volatility modeling of cryptocurrencies using several GARCH models and found Bitcoin, Ethereum, Litecoin, and many others exhibit extreme volatility [36]. Dyhrberg investigated the financial asset capabilities of bitcoin using GARCH models. The overall result of the study suggests that bitcoin is somewhere in between a currency and a commodity due to its decentralized nature and limited market size [37].

Several approaches have been employed to examine the interrelationship between Bitcoin and other cryptocurrencies. Yi et al. used the spillover index approach and its variants to investigate static and dynamic volatility connectedness among eight typical cryptocurrencies. The results reveal that their connectedness fluctuates cyclically [38]. Ciaian et al. empirically examined interdependencies between Bitcoin and altcoin markets for the short and long-run periods using the time series analytical mechanisms. The results of the study show that the Bitcoin-altcoin price relationship is stronger in the short-run than in the long-run [39]. Katsiampa studied the co-movement of volatility between Bitcoin and Ether using a Diagonal BEKK model and found the two cryptocurrencies’ volatility and correlation are responsive to the big news [40]. Recently, Assaf et al. employed transfer entropy and Rényi’s transfer entropy to quantify the information flow between Bitcoin, Ripple, and Ethereum and revealed that Bitcoin and Ripple share a bidirectional information transmission. However, no nonlinear information transmission exists according to Rényi’s measure [41]. Assaf et al. also used the mutual information approach to investigate the information sharing between cryptocurrencies during the COVID-19 crisis. [42]. García-Medina and González Farías García-Medina and González Farías, employed transfer entropy as a variable selection methodology for cryptocurrencies using a high-dimensional predictive model framework. In this study, Symbolic estimation of Transfer Entropy (STE) for simulated studies shows better performance when compared to the Granger causality approach when considering a nonlinear and a linear system with many drivers [43]. Overall, these studies support interdependencies between Bitcoin and other cryptocurrencies. The results are dependent on the size of the dataset and methodologies. However, methods based on information-theoretic measures, such as quantification of bi-directional mutual information in the cryptocurrency market, are relatively at an early stage. Previous studies mostly used parametric methods with some assumptions for measuring financial dependency among financial assets. Compared with other methods, transfer entropy has many advantages, such as: (i) it is suitable for both linear and nonlinear dependence, (ii) it does not make any assumption on the underlying relationship of the variables, and (iii) it is completely data-driven.

### 1.4. Hypothesis Development

Cryptocurrency markets are highly volatile, and a significant number of drawdowns are continuously expected in a short period. Therefore, at this point, it is vital to explore the dynamics of volatilities in Bitcoin and other related cryptocurrencies. For this purpose, we use datasets of five cryptocurrencies: Bitcoin (BTC), Ethereum (ETH), Binance coin (BNB), Ripple (XRP), and Litecoin (LTC). All these cryptocurrencies experience high fluctuations in their valuation. The existence of the causal relationship between estimated volatilities of the underlying cryptocurrency datasets can guide investors, risk managers, and policymakers to investigate the dominance of a particular digital currency. Therefore, we quantify the nonlinear dynamics and bi-directional information flow between the five cryptocurrencies. We use (i) the Hurst exponent to study the nonlinear dynamics and existence of long-memory; (ii) investigation of mutual information gained due to the dependencies exposed by log returns, computed realized volatilities for several time windows, and OHLC volatility estimates; and finally, (iii) we aim to examine the directional flow of closing returns and estimated Open-High-Low-Close (OHLC) volatilities of underlying data series. The rich investigation based on Close-to-Close, Garman and Klass [44], Parkinson’s [45], Rogers and Satchell [46], and Garman–Klass and Yang–Zhang [47] OHLC volatilities estimates have not been conducted on highly volatile cryptocurrency data. We investigate the bi-directional flow of information between OHLC volatilities of five cryptocurrencies using the Shannon transfer entropy (STE) and effective transfer entropy (ETE), and Rényi’s transfer entropy (RTE) and establish the linkage between estimated OHLC volatilities.

We notice that significant literature exists to examine the flow of information between financial asset prices based on the transfer entropy method. However, no studies have been conducted to uncover the information flow between the estimated volatilities series of highly volatile datasets based on OHLC estimators. We aim to fill the existing research gap using cryptocurrency datasets. To the best of our knowledge, this is the first study to compute the mutual information of realized volatilities and transfer entropies of OHLC volatility estimators for correlated cryptocurrency datasets. For this purpose, we closely understand the volatility dynamics of underlying datasets using different volatility estimators based on methods of non-linear complexity measures. Therefore, we have focused on the evaluation of the nonlinear statistics such as the Hurst exponent [48], mutual information (MI) [49], transfer entropy (TE) [13], and effective transfer entropy (ETE) [14] methods to detect the directional information flow. Given the preceding discussion and arguments, we set the following hypothesis.

**Hypothesis** **1** **(H1).** *Bitcoin (BTC), Ethereum (ETH), Binance coin (BNB), Ripple (XRP), and Litecoin (LTC) log returns and estimated OHLC volatilities exhibit long memory*.

**Hypothesis** **2** **(H2).** *BTC, LTC ETH, BNB, and XRP log returns, estimated realized volatilities and estimated OHLC volatilities share mutual information flow*.

**Hypothesis** **3** **(H3).** *Bitcoin and other cryptocurrencies can act as an influencer of price and volatility changes in other cryptocurrencies based on bi-directional information flow*.

The rest of the paper is organized as follows. Section 2 presents methodologies of measuring the statistical complexity and information flows. In Section 3, we analyzed our datasets. In Section 4, empirical results are discussed. Finally, conclusions are summarized in Section 5.

## 2. Methods

### 2.1. Returns, Volatility and Correlation

Let $R_{t}$ denote the returns of the cryptocurrency financial time series process at time *t* given by

(1) $$R_{t}=\ln\frac{x_{t}}{x_{t-1}}$$

where ${\left\{X_{t}\right\}}_{t\;\in T}$ represents the underlying price process. Statistically, volatility is often computed as the sample standard deviation given by

(2) $$S=\sqrt{\frac{1}{T-1}\overset{T}{\underset{t-1}{\sum }}{\left(R_{t}-\mu \right)}^{2}}$$

where μ is the average return over the period *T*. The historical volatility of the annual logarithmic returns is customarily denoted by σ and computed by the following equation:

(3) $$\sigma =S\sqrt{\Delta t}$$

where *t* denotes annual trading days, usually. The estimated historical volatility σ captures only linear relationships and assumes all events are equally weighted. In financial markets, volatility estimates based on a small dataset might lead to noisy measurement of the estimator due to sampling error. On the other hand, a large dataset uses information that is no longer relevant to the present state of the market. A historical volatility estimate is an exact number that may not represent the actual value. To address the problem of large-sampling error, alternative volatility estimators that use all data points other than closing prices, such as Close-to-Close, Garman and Klass (GK), Parkinson’s, Rogers and Satchell (RS), and Garman and Klass–Yang and Zhang (GK-YZ), are given by the following equations, respectively.

(4) $$\sigma_{\mathrm{Close}-\mathrm{to}-\mathrm{Close}}=\sqrt{\frac{N}{n-2}\overset{n-1}{\underset{k=1}{\sum }}{\left(R_{k}-\bar{R}\right)}^{2}}$$

where $R_{k}=\log\left(\frac{C_{k}}{C_{k-1}}\right)$, $\bar{R}=\frac{R_{1}+R_{2}+.\;.+R_{k-1}}{k-1}$ and $C_{k}$ denotes the kth closed price, n is the number of periods for the volatility estimate, N is number of periods per year. Close-to-Close volatility estimator only requires us to look at closing prices and use long historical data for a better estimate. In such a long data series, the earlier part of the data may be less relevant to measuring today’s volatility. Therefore, the usage of alternative estimators of volatility can be more efficient than Close-to-Close.

The Parkinson volatility estimator is based on high and low prices given by:

(5) $$\sigma_{\mathrm{Parkinson}}=\sqrt{\frac{1}{4n\ln2}\overset{n}{\underset{k=1}{\sum }}{\left(\log\frac{H_{k}}{L_{k}}\right)}^{2}}$$

The Parkinson estimator is about five times more efficient than the Close-to-Close estimator. The Parkinson estimate of variance is unbiased for continuous prices. However, prices are only sampled discretely. Another well-known volatility estimator introduced by Garman and Klass (GK) is given by:

(6) $$\sigma_{\mathrm{GK}}=\sqrt{\frac{N}{n}\sum \left[\frac{1}{2}{\left(\log\frac{H_{k}}{L_{k}}\right)}^{2}-\left(2\log2-1\;\right){\left(\log\frac{C_{k}}{O_{k}}\right)}^{2}\right]}$$

where $O_{k}\;,H_{k}$, and $L_{k}$ denote *k*th Open, High, and Low prices. The Garman and Klass volatility estimator assumes Brownian motion with zero drift and no opening jumps. The GK estimator is 7.4 times more efficient than the Close-to-Close estimator, but it is biased due to discrete sampling. Rogers et al. (RS) improved the efficiency of the volatility estimator by introducing a drift term that outperformed. The volatility estimator is given by:

(7) $$\sigma_{\mathrm{RS}}=\sqrt{\frac{N}{n}\sum \left[\log\frac{H_{k}}{C_{k}}\;\times \log\frac{H_{k}}{O_{k}}+\log\frac{L_{k}}{C_{k}}\times \log\frac{L_{k}}{O_{k}}\right]}$$

Recently, Yang and Zhang (YZ-GK) developed a modified version of the Garman and Klass estimator that allows opening jumps. It is a weighted average of the RS estimator, Close-to-Open volatility, and Open-to-Close volatility. The estimator is given by:

(8) $$\sigma_{\mathrm{GK}-\mathrm{YZ}}=\sqrt{\frac{N}{n}\sum \left[\;{\left(\log\frac{O_{k}}{C_{k-1}}\right)}^{2}+\frac{1}{2}{\left(\log\frac{H_{k}}{L_{k}}\right)}^{2}-\left(2\log2-1\right){\left(\log\frac{C_{k}}{O_{k}}\right)}^{2}\right]}$$

All these volatility estimators on simulated discretely sampled with drift and jumps show a high correlation between estimators. Pearson’s correlation is one of the simple dissimilarity measures which can be used to measure the correlation. For time series observation, the measure is given by:

(9) $$\mathrm{Corr}\left(X_{T},Y_{T}\right)=\frac{\sum_{t=1}^{T}\left(X_{t}-\bar{X_{T}}\right)\left(Y_{t}-\bar{Y_{T}}\right)}{\sqrt{\sum_{t=1}^{T}{\left(X_{t}-\bar{X_{T}}\right)}^{2}}\sqrt{\sum_{t=1}^{T}{\left(Y_{t}-\bar{Y_{T}}\right)}^{2}}}$$

where ${\left\{X_{t}\right\}}_{t\;\in T}$ and ${\left\{Y_{t}\right\}}_{t\;\in T}$ represent the underlying price processes.

### 2.2. Hurst Exponent

The Hurst exponent is a tool for studying several properties of a time series. The method was first introduced by Hurst (1951) and is known as Rescaled Range Analysis (R/S analysis) [48]. In financial statistics, it is used for the analysis of stochasticity, fractality, volatility shifts, and long-run memory of a time series. The Hurst model is based on the work of Albert Einstein on the Brownian motion of random particles. The essence of the model follows a series of observations (samples) and calculates a distance *R* that increases in proportion to the square root of the time *T*. Let a series of observation of prices $X_{1},\;X_{2},\ldots ,X_{N}$ of a fixed length *N*. The Hurst exponent is given by:

(10) $$\frac{R}{S}=k.N^{H}$$

- where *R* denotes the rescaled range of variation, *S*—standard deviation, *k*—constant, *N*—number of sample elements, *H*—the Hurst exponent;
- The hurst exponent ranges from zero to one;
- For random (Wiener) processes in particular, the Hurst index turns out equal to 0.5;
- A value larger than 0.5 may indicate a fractal model or long-run dependence and positively correlated;
- A value less than 0.5 indicates rough anti-correlated series.

The rescaled range (R/S) analysis is the range of partial sums of deviations from their means, which is rescaled by their standard derivations. The computation Algorithm 1 of the Hurst exponent using R/S analysis comply as:
**Algorithm 1**. Hurst Exponent Computations1.Suppose the given time series has length N. In the first step, convert the original time series to a log return series;2.Divide the entire data series into “*A*”, several contiguous sub-periods $I_{a}\;$, with a=1,2,…,A. Each element in $I_{a}$ is labeled as $N_{k,\;a}$, where k=1,2,…,n. For each $I_{a}$, compute the average $e_{a};$3.Create a series of accumulated departures $D_{k,\;a}$ from the mean value for each sub-period by defining $D_{k,\;a}=\overset{k}{\underset{i=1}{\sum }}\left(N_{i,\;a}-e_{a}\right)$;4.Define the range as maximum minus the minimum value of accumulated departures within each sub-period by $R_{I_{a}}=\max\left(D_{k,\;a}\right)-\min\left(D_{k,\;a}\right)$;5.Calculate the sample standard deviation $S_{I_{a}}$ of each sub-period $I_{a}$;6.Normalize each range by dividing by the sample standard deviation. Therefore, the rescaled range for each $I_{a}$ is equal to $R_{I_{a}}/S_{I_{a}\;}$;7.Compute the average of ${\left(\frac{R_{I_{a}}}{S_{I_{a}\;}}\right)}_{n}=\left(1/A\right)\overset{A}{\underset{a=1}{\sum }}\left(\frac{R_{I_{a}}}{S_{I_{a}\;}}\right)$;8.Following the [48] Hurst exponent can be obtained using Equation (10). Estimate the Hurst exponent by running OLS regression, taking logarithm values of the series.

### 2.3. Shannon Entropy

Shannon (1948) defined a measure of information contained by an experiment in the context of the mathematical theory of communication [50]. The entropy measure has applications in physics, biology, economics, sociology, and other fields. For example, in financial studies, Shannon entropy measures the randomness and diversity of the price’s series. Mathematically, Shannon’s entropy gives the measure of information as a function of probabilities of occurrence of different random events. The Shannon entropy of the random variable having the discrete probability distribution is defined by:

(11) $$H\left(X\right)=-\overset{m}{\underset{i=1}{\sum }}p_{i}\ln p_{i}$$

- where $X=\left\{x_{1,}x_{2,},\ldots ,x_{m,}\right\}$ the convention 0ln0=0 holds, and $p=\left(p_{1},p_{2},\;\ldots ,p_{m}\right)$, $p_{i}$ represents the probability of $x_{i}$, for i=1,2, …,m. therefore,$\;p_{i}>0\;\forall \;i$ and $\overset{m}{\underset{i=1}{\sum }}p_{i}=1$;
- The entropy will be equal to its maximum value if all events follow the equally likely assumption;
- An event for which probability is less than one, the entropy has a positive sign;
- Shannon entropy is utilized for quantifying the variability in an individual random variable.

The Shannon entropy can be used in particular manners to evaluate the entropy corresponding to a probability density distribution around some points. Alternatively, sometimes specific events of interest are significantly important. For example, the deviation from the mean, a piece of sudden news, affecting the market. At this point, the generalization of the classical concept of entropy is vital.

### 2.4. Rényi Entropy

Rényi’s information measure was introduced by Rényi in his seminal 1961 paper [51]. Rényi measure depends on the power of the probability law, and it is a one-parameter generalization of Shannon’s entropy. For a discrete random variable *X*, the Rényi entropy of a probability distribution function *P* is given by:

(12) $$H_{r}\left(X\right)=\frac{1}{1-r}\log_{2}\underset{x\in X}{\sum }p^{r}\left(x\right)$$

- The Rényi’s entropy is a non-negative, monotonically decreasing function of *r* and for *r* = 1, Rényi’s entropy converges to Shannon’s entropy;
- For *r* closer to 0, Rényi entropy becomes uniform to all possible events and independent of the density function of the random variables;
- The different values of *r* can be used to express the influence of the different probability intervals on the results;
- For *r* > 1, the Rényi entropy depends more on the values with large probabilities and less on those of the rare ones.

### 2.5. Mutual Information

The Shannon entropy combined with the concept of Kullback–Leibler divergence allows to measure the information flow between two processes. The mutual information measures the deviation from the independence of the two random variables. Mutual information (MI) is one of the fundamental information-theoretic measures to quantify the information flow between the two data sets *X* and Y. The MI is given by:

(13) $$\mathrm{MI}\left(X,Y\right)=\underset{x\in X}{\sum }\underset{y\in Y}{\sum }p\left(x,y\right)\log\frac{p\left(x,y\right)}{p\left(x\right)p\left(y\right)}$$

The MI represents the divergence between the joint distribution p(x,y) of variables *x* and *y* and the product p(x)p(y) of the two marginal distributions. The MI is a symmetric quantity and can be rewritten as a sum and difference of Shannon entropies given by:

(14) MI(X,Y)=H(X)+H(Y)−H(X,Y)

where H(X,Y) denotes the joint Shannon entropy. The MI is a special case of a measure called the Kullback–Leibler divergence. We can summarize the characteristics of MI as follows.

- Mutual information measures mutual dependence. In other words, it determines how much information is communicated between two random variables;
- We can use MI to infer about one random time series by observing another random one;
- MI measures linear and nonlinear dependencies between two time series. The measure can be used as a nonlinear equivalent of the correlation function;
- It is a symmetric measure, therefore the direction of information cannot be distinguished;
- Higher values indicate stronger dependency, and low values, a weaker dependence. For two independent variables, the MI value is zero.

More details on mutual information can be found in [49].

### 2.6. Shannon and Rényi Transfer Entropies

The concept of transfer entropy (TE) was introduced by Schreiber [13], and independently under the name conditional mutual information by Paluš et al. [12]. TE computes a directional information flow defined by means of Kullback–Leibler divergence on conditional transition probabilities of two times series. Suppose *X* and Y denote two Markov processes. The following two equations define the TE from *X* to Y and Y to *X*, respectively.

(15) $${\mathrm{TE}}_{X\to Y}=T\left(Y_{i+1}|Y_{i}^{u},X_{i}^{v}\right)=\underset{y_{i+1},y_{i}^{u},x_{i}^{v}}{\sum }p\left(y_{i+1},y_{i}^{u},x_{i}^{v}\right)\log_{2}\frac{p\left(y_{i+1}|y_{i}^{u},x_{i}^{v}\right)}{p\left(y_{i+1}|y_{i}^{u}\right)}$$

(16) $${\mathrm{TE}}_{Y\to X}=T\left(X_{i+1}|X_{i}^{u},Y_{i}^{v}\right)=\underset{x_{i+1},x_{i}^{u},y_{i}^{v}}{\sum }p\left(x_{i+1},x_{i}^{u},y_{i}^{v}\right)\log_{2}\frac{p\left(x_{i+1}|x_{i}^{u},y_{i}^{v}\right)}{p\left(x_{i+1}|x_{i}^{u}\right)}$$

where *u* and *v* are Markov orders, and $x_{i}^{v}=\left\{x_{i},\ldots ,x_{i-v+1}\right\}$ and $y_{i}^{u}=\left\{y_{i},\ldots ,y_{i-u+1}\right\}$ are past states. In simple manners, TE can be described as the mutual information (MI) between the future of Y and the present of X once conditioned to the past of Y.

(17) $$\mathrm{TE}\left(X\to Y\right)=\mathrm{MI}(Y^{+};X^{-}|Y^{-})$$

where the superscripts + and − denote adequate future and past state reconstructions of the respective random variables. Equation (17) enables transfer entropy (i) to consider the transition between states and thus incorporates the dynamics of the processes, and (ii) transfer entropy is inherently asymmetric with respect to the exchange of X and Y and thus can distinguish the two possible directions of interaction [52]. Therefore, Equation (17) can be rewritten as:

(18) $$\mathrm{TE}\left(X\to Y\right)=\mathrm{MI}\left(Y^{+};\left(X^{-},Y^{-}\right)\right)-\mathrm{MI}\left(Y^{+};Y^{-}\right)$$

We summarize the TE and its fundamental properties as follows.

- The TE is defined as the ratio of the conditional distribution of one variable depending on the past samples of both processes versus the conditional distribution of that variable depending only on its own past values;
- The asymmetry of TE results in a differentiation of the two directions of information flow;
- We may note that for independent processes, the TE is zero and it is not a symmetric measure;
- The TE quantifies the information flow from process *X* to process Y by measuring the deviation from the generalized Markov property;
- The difference between ${\mathrm{TE}}_{X\to Y}$ and ${\mathrm{TE}}_{Y\to X}$ allows to discover the dominant direction of the information flow;
- The common choices of the order of the Markov process are conducted by u=v=1;
- Transfer entropy (TE) is closely related to conditional entropy, but it extends to two processes.

### 2.7. Effective Transfer Entropy

The actual data from financial time series often contained noise, which may bring uncertainty. Therefore, to solve this problem, Marschinski and Kantz modified the transfer entropy known as effective transfer entropy (ETE) [14]. The effective transfer entropy can be formulated as follows.

(19) $${\mathrm{ETE}}_{Y\to X}\left(u,v\right)={\mathrm{TE}}_{Y\to X}\left(u,v\right)-{\mathrm{TE}}_{S\to X}\left(u,v\right)$$

where ${\mathrm{TE}}_{S\to X}$ represents the average of TE from the shuffled sequence to responding sequence. The modified approach negates the statistical dependencies between the two processes Y and *X* and the dependencies of process *Y*. Indeed, a new time series was generated by a readjustment of randomly drawing values from process Y. This approach could reduce the noise caused by random process. As a consequence, ${\mathrm{TE}}_{S\to X}\left(u,v\right)$ converges to zero when the sample size increases and its values different from zero are due to small sample effect. In calculating the effective transfer entropy, commonly shuffling the series many times and using the transfer entropy estimate averaged over the replications have been used. We assess the statistical significance of the estimated transfer entropies based on n times bootstrapping of the Markov process. Such an approach preserves the dependencies within the variables u and v but eliminates the statistical dependencies between them. Given bootstrapped distribution of the transfer entropy estimates, the dominant direction of the information flow can be confirmed by deriving standard errors and *p*-values for the effective transfer entropy [22].

Jizba and Kleinert proposed Rényi transfer entropy (RTE) [21] based on Rényi entropy [51] and transfer entropy [13]. The RTE is given by:

(20) $${\mathrm{RTE}}_{Y\to X}=\frac{1}{1-r}\log_{2}\frac{\sum_{x_{i+1},x_{i}^{u}}\phi_{r}\left(x_{i}^{u}\right)p^{r}\left(x_{i+1}|x_{i}^{u}\right)}{\sum_{x_{i+1},x_{i}^{u},y_{i}^{v}}\phi_{r}\left(x_{i}^{u}\right)p^{r}\left(x_{i+1}|x_{i}^{u},y_{i}^{v}\right)}$$

The trend of the entropy is irregular concerning *r*. That means some sequence groups show an upward trend with the increased *r*, while others show a downward trend. In general, the method is comprehensive. Therefore, for a clear direction of the information flow, one may perform many experiments. Effective Rényi transfer entropy (ERTE) can be obtained by following the same logic as ETE [14]. The ERTE is given by [53]:

(21) $${\mathrm{ERTE}}_{Y\to X}\left(u,v\right)={\mathrm{RTE}}_{Y\to X}\left(u,v\right)-{\mathrm{RandomRTE}}_{S\to X}\left(u,v\right)$$

where ${\mathrm{RandomRTE}}_{S\to X}$ represents the average of RTE from the shuffled sequence to responding sequence. In summary, the following Algorithm 2 is required to compute the transfer entropies on statistical software such as R.**Algorithm 2**. Transfer Entropies Computations1.Select two vectors of numeric values X and Y ordered by time;2.Select Markov orders of X and Y. The default value is one;3.Specify the transfer entropy (TE) measure to be estimated;4.Choose the number of shuffles used to calculate the effective transfer entropy (ETE). The default is 100;5.Specify the type of discretization: “quantiles”, “bins” or “limits”;6.Specify quantiles of empirical distribution. Default is quantiles = C (5,95) or go to step 7;7.Specify the number of bins with equal width used for discretization. Default is bins = Null or go to step 8;8.Specify the limits on values used for discretization. Default is limit = Null;9.Select number of bootstrap replications for each direction of the estimated transfer entropy. Default is 300;10.Select the number of observations that are dropped from the beginning of the bootstrapped Markov chain. Default is 50;11.Select “quiet” if FALSE (default), the function gives feedback.

## 3. Analyzing Data

### 3.1. Distributional Properties and Nonlinearity Tests

**Figure 1:**
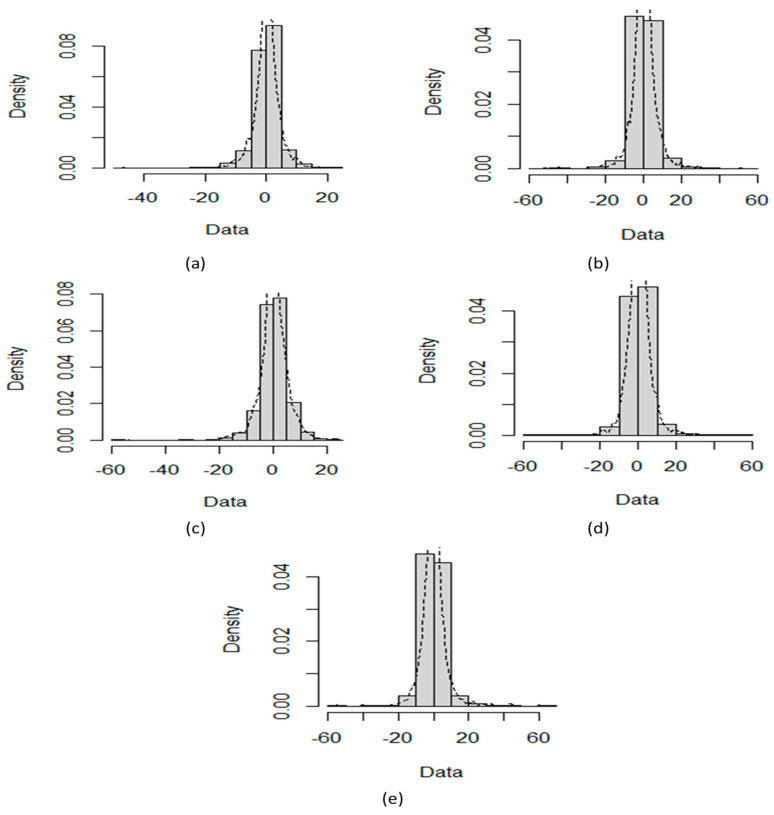
Density plots of percentage log returns of BTC (**a**), LTC (**b**), ETH (**c**), BNB (**d**), and XRP (**e**).

**Figure 2:**
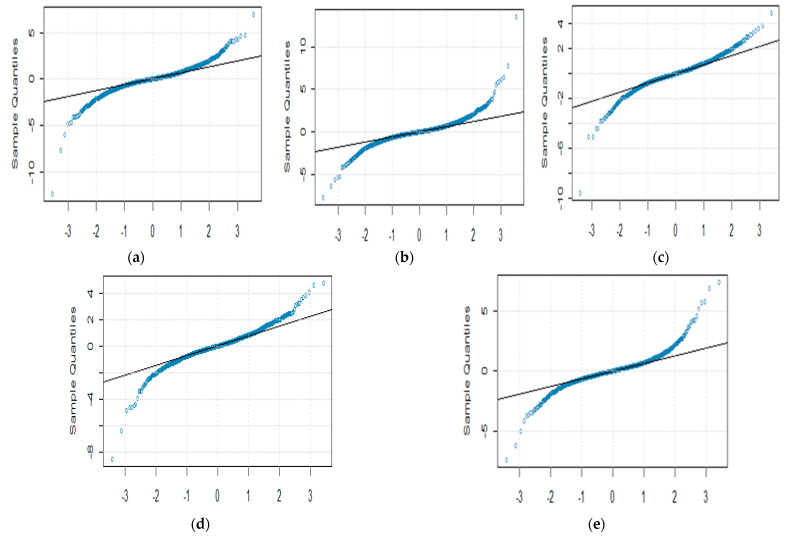
QQ plots of BTC (**a**), LTC (**b**), ETH (**c**), BNB (**d**), and XRP (**e**).

The daily closing prices of five cryptocurrencies, including BTC, LTC, ETH, XRP, and BNB financial time series, are investigated for the mutual relationship. The data used are prices (in US dollars) for all the time series available online at http://finance.yahoo.com, accessed on (10 March 2022). We consider 2730 observations from 18 September 2014 to 9 March 2022 for BTC and LTC, while for XRP, BNB, and ETH, the closing time series follows from 9 November 2017 to 10 March 2022. Similarly, for OHLC volatility estimators, the same cryptocurrencies for the same periods have been used. We compute the logarithmic returns ($R_{t}$) of underlying daily price process ${\left\{X_{t}\right\}}_{t\in \left[0,T\right]}$ by $R_{t}=\log\left(\frac{x_{t}}{x_{t-1}}\right)$ to obtain the stationarity. Figure 1 shows density plots of the five cryptocurrencies. Table 1 shows the basic statistics of log returns series and nonlinearity test results that include the Teraesvirta Neural Network Test (TNNT), White Neural Network Test (WNNT), and Tsay Test (TT). All the *p*-values except WNNT for LTC are less than equal to 0.05, rejecting the null hypothesis of linearity existence. All cryptocurrencies are negatively skewed and show that large negative returns occur more often than large positive ones. The high values of kurtosis indicate the fat-tailed and peaked returns distribution. Therefore, large and small returns come about more often than expected. Figure 2 depicts QQ plots of five digital currencies and shows prominent deviation from the normally distributed returns. Transfer entropy estimation requires the data series to be stationary. Therefore, the augmented Dickey–Fuller test was performed on log returns and OHLC volatility estimates of all cryptocurrencies. Hence, the test results imply the stationarity at the 1% significance level for log returns and OHLC volatility estimates.

### 3.2. Anomalies in Cryptocurrencies Data

**Figure 3:**
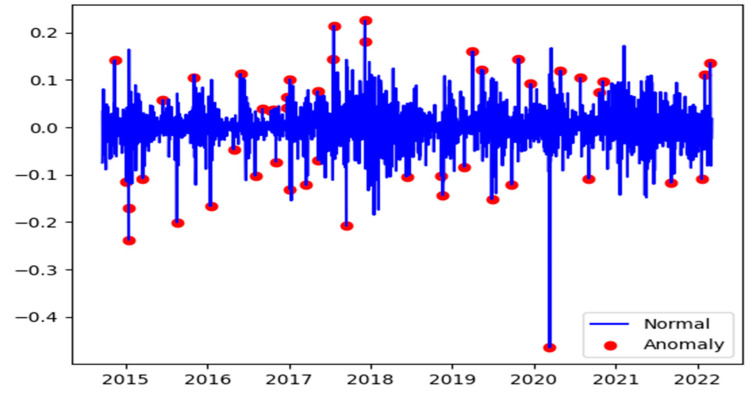
BTC returns series values and anomalies during the period 2014–2022 displaying years on x-axis versus the return values on y-axis.

Cryptocurrencies data are usually highly volatile due to rapid changes in prices, and logarithmic returns might observe the existence of anomalies. For example, Figure 3 shows the returns series plot of the BTC with a red dot marked as an anomaly. We used the 3-sigma approach for each time point and calculated the moving averages and standard deviations using the last one, three, six, nine months, and one year’s trading days to detect the anomalies.

**Figure 4:**
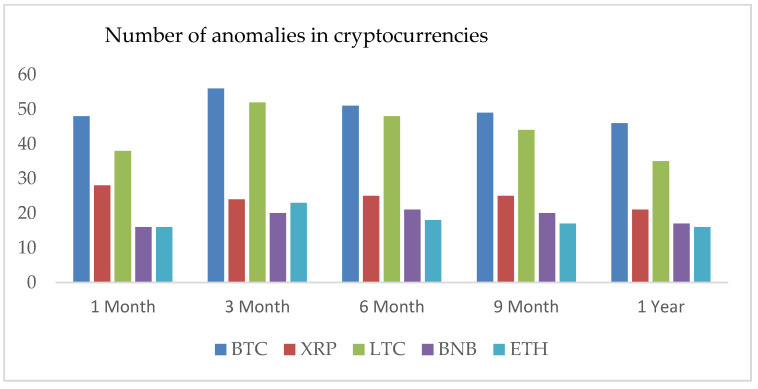
Number of anomalies in all cryptocurrencies for different periods. The years on x-axis versus the number of anomalies on y-axis.

We can observe for each period, LTC exhibits the highest standard deviation of number of anomalies, and BTC follows the second position. However, the rest of the cryptocurrencies have standard deviations closed to each other. See Figure 4. Additionally, we investigate the correlation coefficients (ρ) between the return’s series of BTC and LTC and similarly between XRP, ETH, and BNB that turn out positive for all datasets. BTC and LTC share the highest value of ρ equals to 0.68. On the other hand, ETH-XRP, XRP-BNB, and ETH-BNB, we observe the correlation values are 0.64, 0.49, and 0.65, respectively. We have observed a positive slope of the regression line and a strong positive correlation between BTC and LTC.

### 3.3. Long Memory

**Figure 5:**
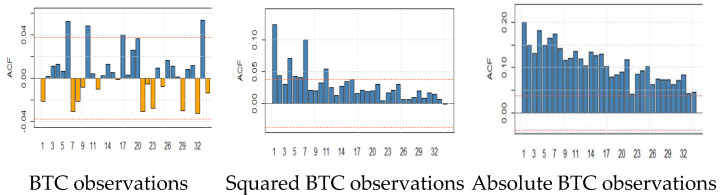

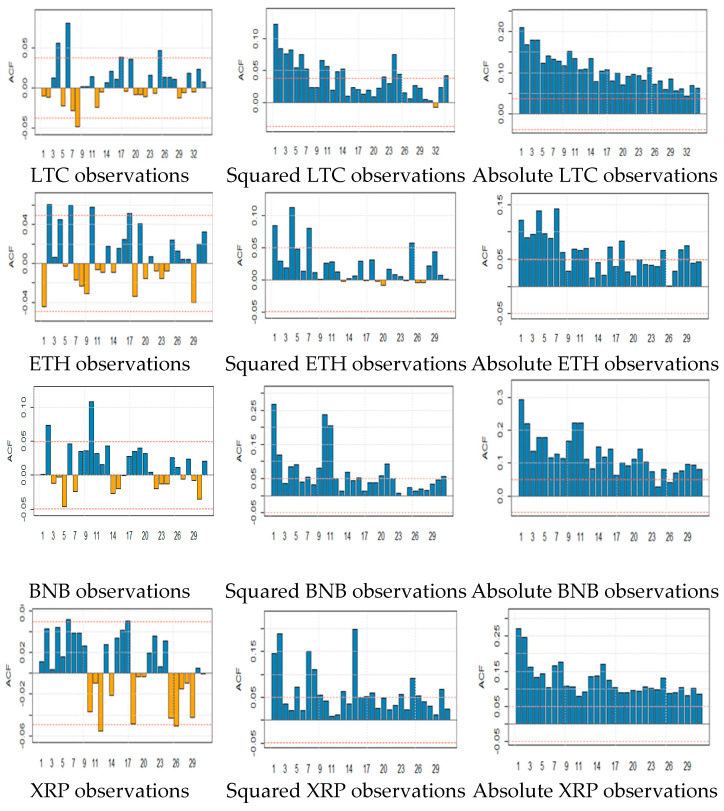
Autocorrelation function (ACF) plots of BTC, LTC, ETH, BNB, and XRP returns, squared, and absolute observations. The x-axis of the ACF plot indicates the lag (up to 29 for each plot) at which the autocorrelation is computed; the y-axis indicates the value of the correlation.

We examine the significance of autocorrelation functions for daily closing returns of five underlying cryptocurrencies. We observe that autocorrelation functions are insignificant for all returns series. Additionally, autocorrelation functions of both squared and absolute returns are significant for almost all lags and decay slowly. For example, the decay in the absolute returns is more prominent and similar in the case of BTC and LTC. The slow decline in autocorrelation functions is named a long memory characteristic. The long memory of returns suggests volatility clustering and volatility of past returns would affect future returns for a long time. Figure 5 depicts the autocorrelation functions of BTC, LTC, ETH, BNB, and XRP.

## 4. Results

### 4.1. Hurst Exponent Analysis

**Figure 6:**
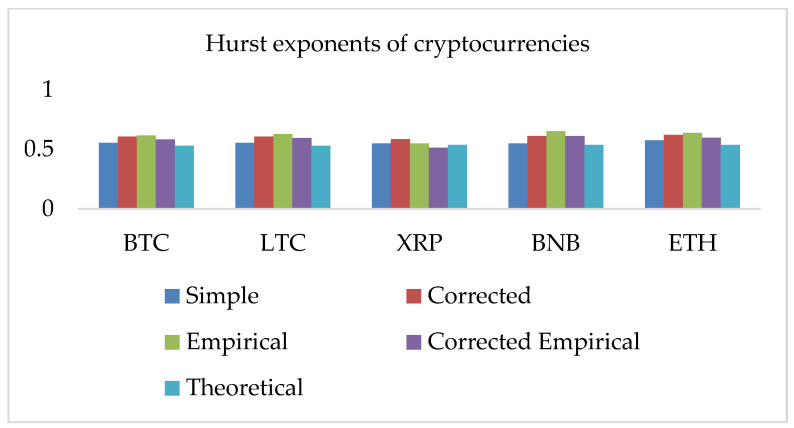
Hurst exponents (HE) of the five selected cryptocurrencies returns series data. The y-axis shows HE values of the corresponding x-axis cryptocurrency.

We have observed a strong positive correlation among the five selected cryptocurrencies. Following the methodology, we investigated (i) the Hurst Exponent (HE) for closing returns of BTC, LTC, XRP, BNB, and ETH. The HE analysis requires large samples. For this reason, we implemented the methodology on daily returns series data of several years. We can observe all computed values of the Hurst exponent (HE) are higher than 0.5, which suggests all returns series exhibit long-run dependence, employment of a fractional model, and positively correlated data. The distribution of the nonlinear complexity measure HE is shown in Figure 6.

**Figure 7:**
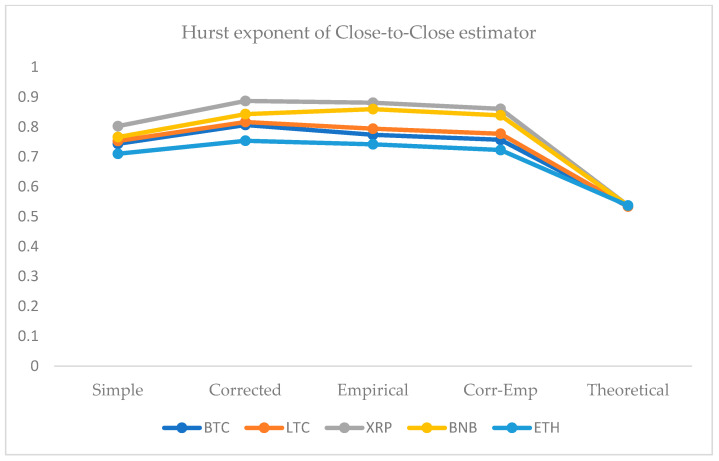
Variations in Hurst exponents (HE) values of estimated OHLC Close-to-Close volatility estimates of BTC, LTC, XRP, BNB, and ETH. The y-axis shows HE values of the cryptocurrency using simple, corrected, empirical, corrected empirical and theoretical methods of HE computation.

**Figure 8:**
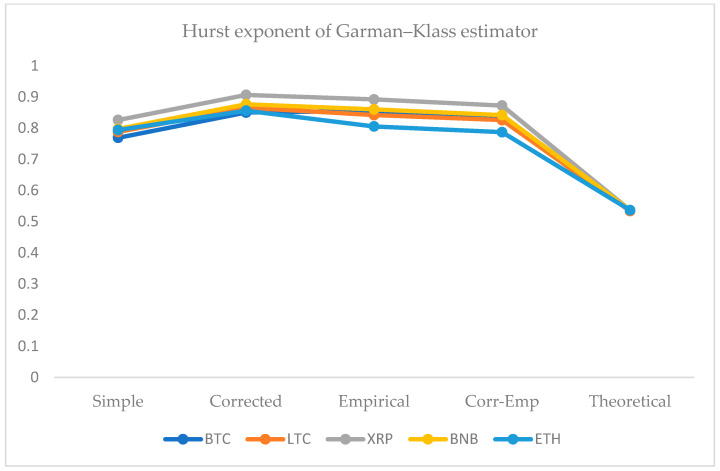
Variations in Hurst exponents (HE) values of estimated OHLC Garman–Klass volatility estimates of BTC, LTC, XRP, BNB, and ETH. The y-axis shows HE values of the cryptocurrency using simple, corrected, empirical, corrected empirical and theoretical methods of HE computation.

**Figure 9:**
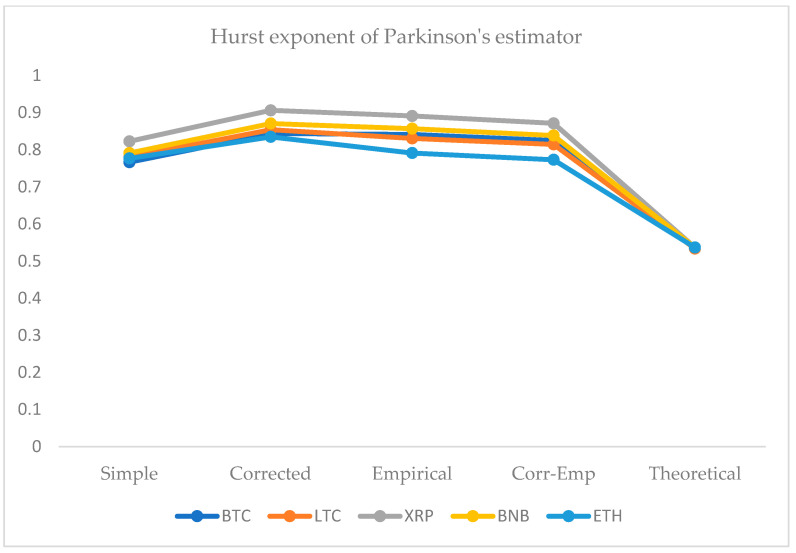
Variations in Hurst exponents (HE) values of estimated OHLC Parkinson’s volatility estimates of BTC, LTC, XRP, BNB, and ETH. The y-axis shows HE values of the cryptocurrency using simple, corrected, empirical, corrected empirical and theoretical methods of HE computation.

**Figure 10:**
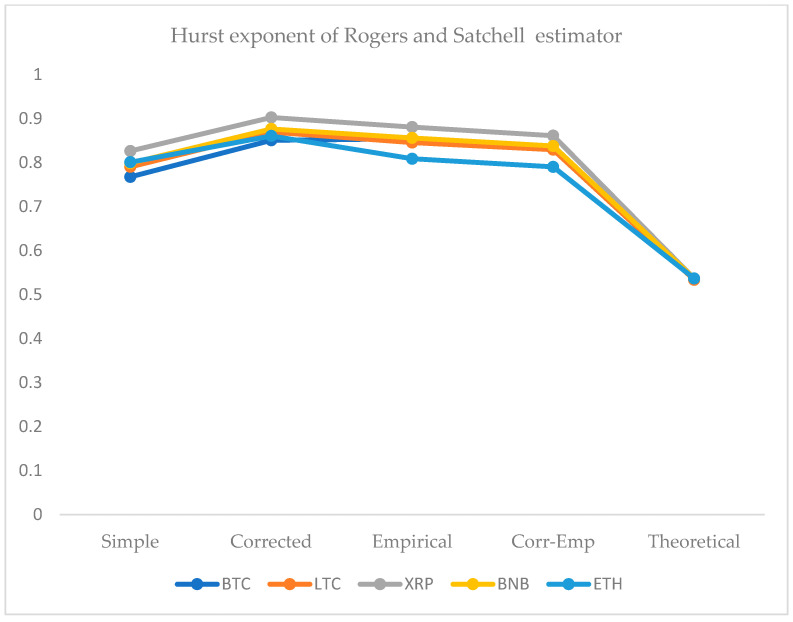
Variations in Hurst exponents (HE) values of estimated OHLC Rogers and Satchell volatility estimates of BTC, LTC, XRP, BNB, and ETH. The y-axis shows HE values of the cryptocurrency using simple, corrected, empirical, corrected empirical and theoretical methods of HE computation.

**Figure 11:**
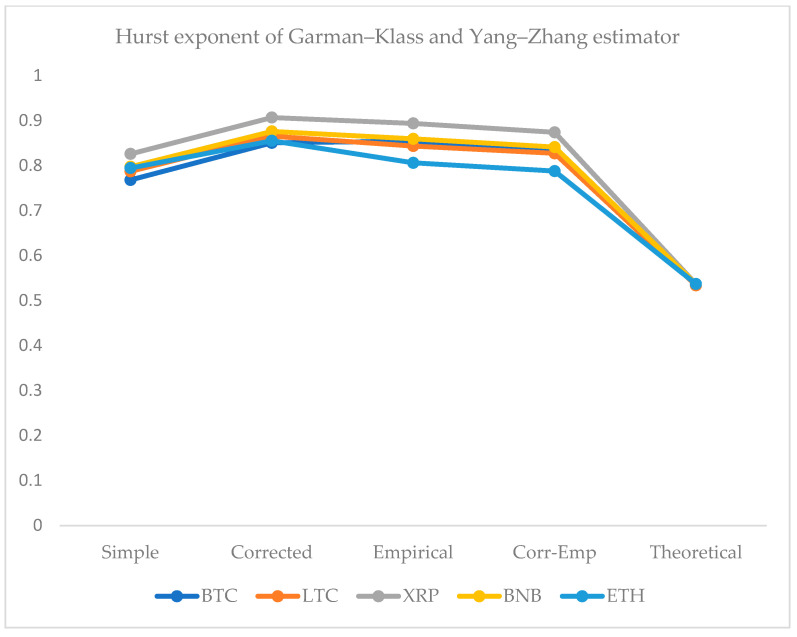
Variations in Hurst exponents (HE) values of estimated OHLC Garman–Klass and Yang–Zhang volatility estimates of BTC, LTC, XRP, BNB, and ETH. The y-axis shows HE values of the cryptocurrency using simple, corrected, empirical, corrected empirical and theoretical methods of HE computation.

We employed five approaches for the computation of the HE. For this purpose, we use the simple R/S, corrected R/S, empirical, corrected-empirical, and theoretical method. We notice that for implemented approaches on returns, the resulted values have a range between 0.55 and 0.65, which characterizes the cryptocurrency data as significantly correlated with long-run memory. Similarly, using OHLC volatility estimators, we compute the volatilities of the BTC and LTC and XRP, BNB, and ETH datasets for the given periods. All estimated values of Hurst exponents are determined by employing five different methods ranging from a minimum value of 0.53 to a maximum of 0.90. Therefore, the volatility dynamics of all cryptocurrencies show long-run dependence and non-linear behavior. Figure 7, Figure 8, Figure 9, Figure 10 and Figure 11 depict the variations of the Hurst exponent for OHLC volatility estimators. A high value of HE closer to 1 indicates an eminent risk of large, exceedingly sudden and unexpected changes in volatility. For example, in the case of BTC, we observed a significant number of anomalies with large volatility jumps. A similar behavior in other cryptocurrencies has been noticed. However, surprisingly, the trend of the Hurst exponent is higher in the case of XRP and lower for ETH. The range of computed HE values recommends that investors and risk managers can expect abrupt changes in volatilities that occur today might impact the future in the long-term. The chaotic nature of volatilities would sensitively depend on initial conditions, and long-memory characteristics might happen regardless of the time scale. We can observe that the results based on the Hurst exponent are compatible with our findings of cryptocurrencies returns characteristics. We have shown that autocorrelation functions of squared and absolute returns of cryptocurrencies have shown slow decayed. It provides a reasonable ground to ensue the existence of long memory.

### 4.2. Mutual Information of Cryptocurrencies Returns and Estimated Volatilities

**Figure 12:**
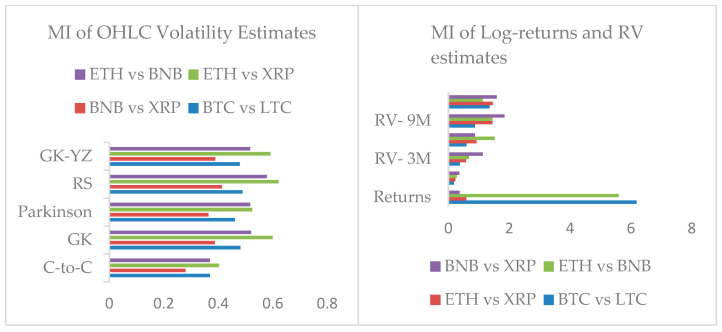
Mutual information (MI) of OHLC volatilities and realized volatilities (RV) estimates for the period one, three, six, and nine months and one year of cryptocurrencies datasets. The y-axis shows computed values of the MI.

We have already observed the existence of correlations between cryptocurrency datasets. Therefore, we compute mutual information gained due to the dependencies exposed by log returns, realized volatilities, and OHLC volatility estimates. BTC and LTC log-returns series gained the highest Mutual-Information (MI), whereas realized volatilities (RV) estimates show an increasing trend of gained MI as the time window jumps from one month to one year. We report the highest mutual information shared between ETH and XRP for all OHLC volatility estimates. Similarly, ETH and BNB stand in the second position, whereas BTC and LTC in third place. However, MI results do not imply a relationship between the causes and effects of underlying observations. It is incapable of classifying the information that is interchanged from shared information. Therefore, we examine transfer entropy estimates to further develop insight into information flow among estimated volatilities. Figure 12 shows a comparison of estimated volatilities and mutual information gain. Our MI results based on log returns, RV, and OHLC volatility estimates provide a solid ground to extend the analysis for bi-directional flow among estimated volatilities datasets.

### 4.3. Transfer Entropies Results of OHLC Volatilities

We examine the TE estimates by dividing the selected cryptocurrencies into two data sets. We include two equidistant spaced time series prices and estimated volatilities of BTC and LTC in the first dataset. Similarly, the second dataset has three other individual cryptocurrencies: XRP, BNB, and ETH. The default value of respective numbers of lags equals 1 for the two underlying data series. However, the choice of lag value for a time series generated by a stochastic process is a crucial task [52]. We investigated Shannon transfer entropy (STE), and effective transfer entropy (ETE) estimates for cryptocurrencies estimated OHLC volatilities of XRP-ETH, ETH-BNB, BNB-XRP, and BTC-LTC. To calculate ETE, the default number of shuffles have set to equal 100. The number of bootstrap replications for each direction of the estimated transfer entropy have set to equal 300. All transfer entropies are estimated, in both directions, i.e., the first data set to the second data set, and vice versa. We know from datasets that all cryptocurrencies are highly volatile. Therefore, we extend our results to further study the behavior of volatilities by using several estimators based on Open, High, Low, and Close (OHLC) prices. For example, Table 2 shows basic statistics of estimated Close-to-Close (C-to-C), Garman and Klass (GK), Parkinson’s, Rogers and Satchell (RS), and Garman–Klass and Yang–Zhang (GK-YZ) volatilities estimators of BTC and LTC. The values in parenthesis describe LTC estimates whereas values without parenthesis show BTC results. The TE values for Close-to-Close, Garman–Klass, and Parkinson’s OHLC volatility estimators from BTC to LTC are statistically significant at 0.1%. In the case of LTC to BTC, the significance ranges between 0.1 and 5% for all OHLC estimators. We can observe the changes in estimates of TE by changing the order of the Markov processes. For example, for OHLC estimators for the flow of information from BTC to LTC in both directions, if we use the order of Markov processes from 1 to 5, then statistical significance decreased, respectively, and became not significant for the chosen orders. The RTE estimates for all OHLC estimates of all cryptocurrencies are not statistically significant.

**Figure 13:**
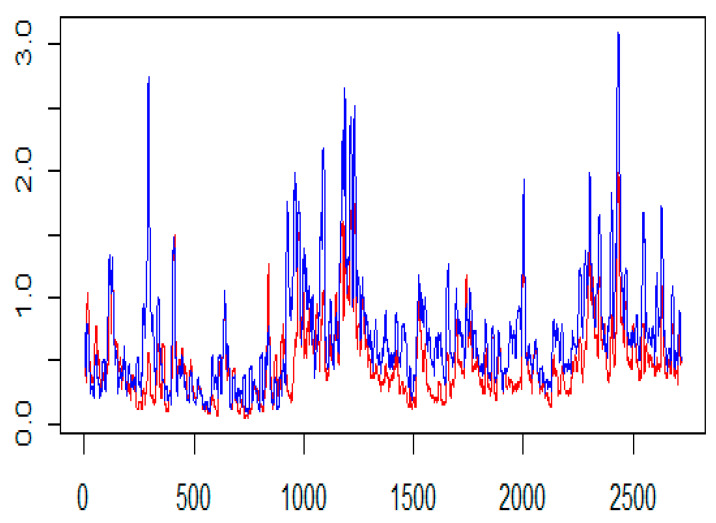
Rogers and Satchell (RS) volatility estimate plots of BTC (red) and LTC (blue). The x-axis shows the number of observations, and the y-axis the corresponding OHLC volatility estimate.

We observe the highest information flow from BTC to LTC and lowest from LTC-BTC for Rogers and Satchell volatility estimates. Figure 13 shows a comparision of Rogers and Satchell volatility estimates. We quantify that transfer entropies for Parkinson’s volatility estimates show significant information in both directions with *p*-values less than 0.001.

**Figure 14:**
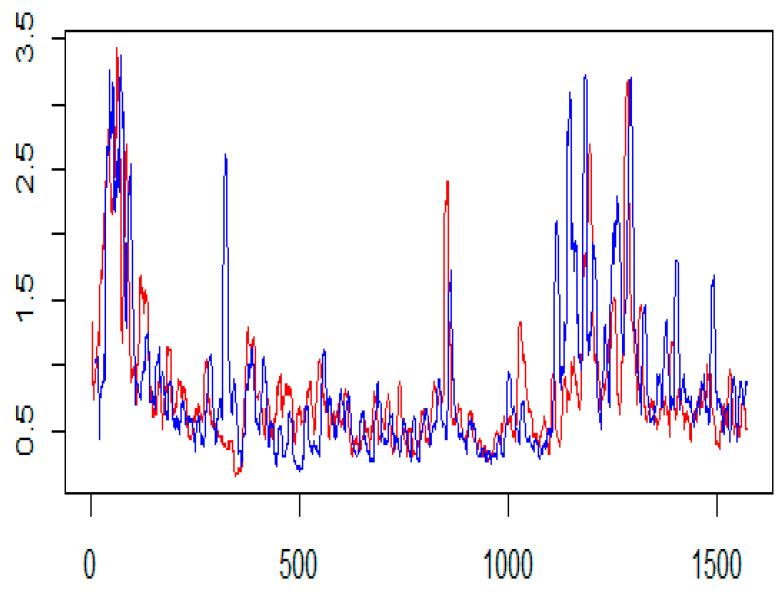
Garmen and Klass (GK) volatility estimates plots of BNB (red) and XRP (blue). The x-axis shows the number of observations, and the y-axis the corresponding OHLC volatility estimate.

**Figure 15:**
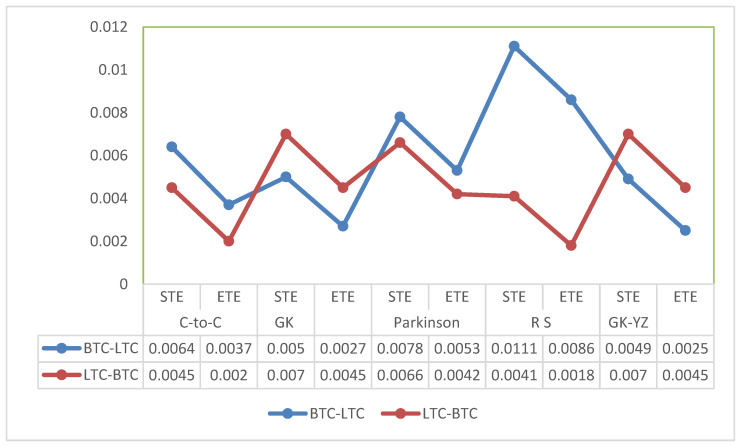

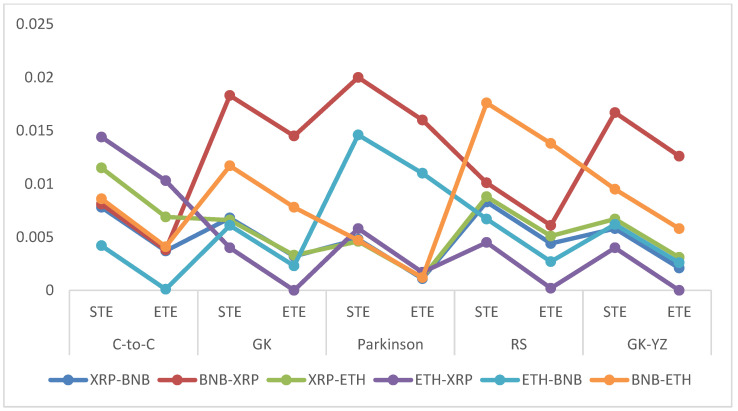
STE and ETE estimates of OHLC volatilities for BTC-LTC (**top**) and XRP, BNB, and ETH (**bottom**) in both directions. The x-axis shows the OHLC estimator, and the y-axis presents corresponding transfer entropy estimates.

**Figure 16:**
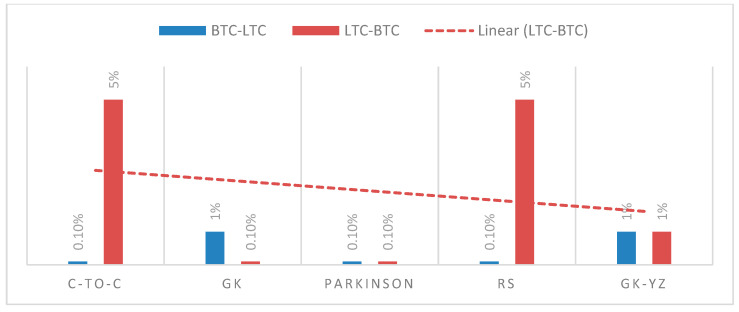
*p*-values of ETE for OHLC volatilities of first dataset of BTC-LTC in both directions. The red dashed line shows trend of the *p*-values from LTC-BTC. The x-axis shows the OHLC estimator, and the y-axis presents significance of the *p*-values.

**Figure 17:**
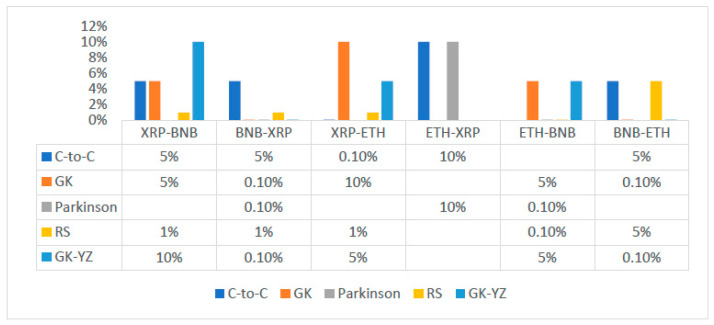
*p*-values of transfer entropy estimates for OHLC volatilities of second dataset (ETH, XRP, and BNB) of cryptocurrencies in both directions. The missing values in the table show TE results are not statistically significant for the respective volatility estimates of the cryptocurrency. The x-axis shows the OHLC estimator, and the y-axis presents significance of the *p*-values.

The STE estimates are highest in the case of BNB to XRP for GK, Parkinson’s and GK-YZ volatility estimators. See, for example, Figure 14. Additionally, all *p*-values are statistically significant, varying from 0.001 to 0.05. The trend of STE and ETE estimates is declining for ETH to XRP. Figure 15 shows a detailed trend analysis of estimates of transfer and effective transfer entropies of all cryptocurrencies. Figure 16 and Figure 17 depict the variations in the corresponding *p*-values of transfer entropy estimates for both datasets, respectively. In table values of Figure 17, missing *p*-values represent that transfer entropies are not statistically significant for the underlying volatility estimates.

## 5. Discussion and Conclusions

Following the efficient market hypothesis, any hidden information cannot be used to predict future market dynamics, and market changes can be represented by a normal distribution [54]. However, we observe cryptocurrency returns dynamics do not follow normality assumption. The heavy-tailed phenomena fit well with cryptocurrency log returns that exhibit higher kurtosis than the normal distribution [55,56,57]. We noticed a prominent count of anomalies developed in all the datasets that oppose efficient market characteristics. In this paper, to investigate and support the long-term existence of memory in cryptocurrency data, in the first step, we performed an analysis based on the computation of Hurst exponents of log returns and OHLC volatility estimates. We summarize our results based on Hurst exponent analysis as follows.

- We conclude that underlying returns and estimated volatilities movements of five cryptocurrencies are not independent over time;
- All datasets contain positive long-term autocorrelation, which implies persistent time series with long-term memory and connection with the Hurst exponent;
- We obtained all HE values larger than 0.5 for all returns series data that indicate a fractal model or long-run dependence. Therefore, these data series might attempt to express a persistent behavior and a nonlinear variance growth;
- A choice of fractional Brownian motion model for underlying data series can incorporate the variance that does not grow linear over time;
- Traditionally, economists investigate and execute analyses under the efficient market hypothesis following the standard Brownian motion model. Our results recommend that the volatility series of these cryptocurrencies tend to grow faster over time because all Hurst exponents are higher than 0.5 for all OHLC estimates;
- The Hurst exponents greater than 0.5 indicates an inefficient market. Therefore, investors, risk managers, and policymakers could distinguish the underlying returns or estimates of volatility series based on the value of the Hurst exponent;
- Our study proposes that log returns and estimated volatility series of Bitcoin and the other four cryptocurrencies deviate from the random walk model and mean reverting characteristics.

Several studies have shown the existence of long memory in cryptocurrency’s returns and volatility. For instance, Soylu et al. tested for the long memory in Ripple, Ethereum, and Bitcoin and uncovered that the squared returns of three cryptocurrencies have a significant long memory [58]. Recently, Wu and Chen have shown that inefficiency and long memory exist in Bitcoin returns over different periods [59]. Lahmiri et al. empirically signify the existence of long-range memory in Bitcoin market volatility by employing the fractionally integrated GARCH (FIGARCH) framework. The results of the study provide strong evidence against the efficient market hypothesis [60]. Rambaccussing and Mazibas examined long memory in cryptocurrencies using other novel tests—the log-periodogram bias test, the skip-sampling test, standard GPH, and local Whittle procedures. The study reports that long memory exists only in Ethereum returns. However, these tests fail to reject the null hypothesis of long memory in most cases across different volatility proxies [61]. In this study, we address and focus on the existence of long memory in five cryptocurrencies using OHLC volatility models. Our results also support the conclusions of previous studies based on different volatility modeling methods. Lastly, for this study, we observed ACF of both squared and absolute returns of all cryptocurrencies are significant for almost all lags and decay slowly. The value of the Hurst exponent is sensitive to the number of time series observations, and its values vary for a shorter and longer data set. We can expect an increase in the Hurst exponent for a large sample, and shorter intervals or high frequencies would anticipate more noise in the data [62].

In the second step, after establishing the existence of long-memory in all cryptocurrencies, we explained the mutual information developed between the log returns, estimated realized volatilities, and OHLC volatility estimates of Garman–Klass, Parkinson’s, Roger–Satchell, and Garman–Klass and Yang–Zhang. Our results show the following facts;

- ETH and BNB, and BTC and LTC shared the highest mutual information;
- For OHLC volatility estimates, ETH and XRP shared the highest mutual information and BTC and LTC show almost a constant trend of sharing mutual information;
- The overall trend of mutual information for realized volatility estimates of BTC and LTC and ETH, XRP, and BNB have increased over time, spanning from one month to one year.

However, mutual information does not provide dynamic and directional information. Therefore, in a third step, we extend our study and report the analysis of the bi-directional flow of OHLC estimated volatilities based on transfer entropy methods. We present empirical results based on OHLC volatilities as follows.

- The Shannon and effective transfer entropies are statistically significant for BTC and LTC in both directions. Similarly, for the second dataset (ETH, BNB, XRP) of underlying cryptocurrencies, all *p*-values for transfer entropies of OHLC estimators are statistically significant;
- Consequently, in the case of transfer entropy estimates of OHLC volatilities, we report the highest information flow from BTC to LTC for Rogers and Satchell. Therefore, BTC is found to be informationally dominant, and extreme changes in BTC volatility should be incorporated consequently into the volatility of LTC;
- We can also examine the net information flow from BTC and LTC. We illustrate from Figure 15 that the net information flow is positive for C-to-C, Parkinson’s and RS estimates, meaning that BTC informationally dominates LTC in most of the OHLC estimates;
- We observed the log returns series of all cryptocurrencies deviate from the normal distribution and exhibit fat-tailed behavior. Consequently, the statistical analysis of estimated OHLC estimates describes rightly skewed volatility distributions. For example, Table 2 shows a case of BTC and LTC following high kurtosis and skewness values of LTC and supports the fat-tailed characteristics. Thus, the data in the distribution tails has extreme relevance, and computation of information flow between volatilities of digital currencies has provided an insight to assess the dominance of underlying digital currency;
- Similarly, for BNB and XRP, the net information flow is positive for all volatility estimates, and BNB prevails over the XRP and ETH in the sense of information flow;
- We conclude that the null hypothesis of no information flow between the estimated volatilities of BTC and LTC and ETH, BNB, and XRP can be rejected at any statistical significance level. However, the TE results depend on the choice of the number of bins into which a given dataset is partitioned and, on the block-length chosen for the transferee and transferor variable.

In this paper, we proposed the employment of different OHLC volatility estimators for cryptocurrency data to quantify the flow of information. We believe the practicable addition of open, high, low, and close volatility estimators for quantifying the transfer entropies between highly correlated datasets will provide an additional choice to compare the results with other existing volatility estimators such as stochastic volatility models. We conclude our results that the volatilities of all datasets show a behavior change over time and fractal and disordered characteristics. The OHLC estimates of volatilities shared correlated nature. Our results are also coloborating with the previous literature on information flow between log returns of cryptocurrencies and other financial time series. For example, recently, Tong et al. [9] also studied Bitcoin price dynamics and showed fractal and chaotic characteristics along with long-memory behavior and a positively correlated nature. Additionally, Assaf et al. [41,42] and Keskin and Aste [63] used information transfer between time series using transfer entropy methods to detect the bi-directional flow between BTC and other cryptocurrency’s prices. Our results are compatible with the ones presented in the previous studies and obtained with different econometric methods. See, for example, [38,39,64,65]. In financial literature, researchers have studied information systems and the effects of bad news on stock price crashes for early warning to prevent the risk of bankruptcy [66,67]. Therefore, our results would be practicable for investors, risk managers, and policymakers to understand the investment strategies based on information flow dynamics of volatilities.

## Figures and Tables

**Table 1:** Basic statistics and nonlinearity test results of cryptocurrencies.

	BTC	XRP	LTC	BNB	ETH
Mean	−0.03703	−0.06191	−0.05493	−0.05214	−0.05728
S.D.	0.07238	0.10920	0.09316	0.09821	0.09256
Skew	−1.26871	−0.64820	−0.69160	−1.16927	−1.19954
Kurtosis	5.9159	7.49848	6.96313	6.86183	5.39491
TNNT	0.00294	0.00000	0.000152	0.000152	0.001269
WNNT	0.00365	0.02157	0.1136	0.00003	0.02069
TT	0.00627	0.00000	0.00000	0.00000	0.00009

**Table 2:** Basic statistics results of OHLC volatility estimators for BTC and LTC in parentheses.

Estimator	C-to-C	GK	Parkinson	RS	GK-YZ
No. Obs	2722	2722	2722	2722	2722
Min.	0.034882 (0.047521)	0.056787 (0.089142)	0.058855 (0.081461)	0.052196 (0.089221)	0.056877 (0.091779)
Max.	2.922745 (3.717033)	1.906995 (3.079054)	2.135686 (3.058603)	1.985108 (3.093214)	1.907693 (3.080433)
Q1	0.309654 (0.411025)	0.289040 (0.711020)	0.304833 (0.412632)	0.278607 (0.403002)	0.289474 (0.410253)
Q2	0.677364 (0.959929)	0.623378 (0.613357)	0.645079 (0.900679)	0.622717 (0.858271)	0.624116 (0.869989)
Mean	0.538511 (0.758029)	0.499735 (0.711020)	0.516278 (0.731707)	0.494480 (0.701918)	0.500892 (0.712596)
Median	0.477780 (0.663103)	0.437840 (0.613357)	0.453897 (0.637059)	0.421143 (0.597912)	0.438332 (0.615175)
SD	0.328704 (0.509606)	0.306106 (0.450409)	0.303292 (0.458338)	0.319009 (0.460012)	0.306424 (0.450420)
Skew	1.833196 (0.509606)	1.476352 (1.778702)	1.435663 (1.735067)	0.319009 (1.843520)	1.471404 (1.780488)
Kurtosis	6.790848 (5.644189)	2.748862 (4.488842)	2.987795 (4.408125)	3.082362 (4.589814)	2.727228 (4.489153)

## Data Availability

Not applicable.
